# Human Sialome and Coronavirus Disease-2019 (COVID-19) Pandemic: An Understated Correlation?

**DOI:** 10.3389/fimmu.2020.01480

**Published:** 2020-06-23

**Authors:** Daniela Morniroli, Maria Lorella Giannì, Alessandra Consales, Carlo Pietrasanta, Fabio Mosca

**Affiliations:** ^1^Fondazione IRCCS Ca' Granda Ospedale Maggiore Policlinico, NICU, Milan, Italy; ^2^Department of Clinical Sciences and Community Health, University of Milan, Milan, Italy

**Keywords:** COVID-19, SARS-CoV-2, human sialome, sialic acid, sialoquake, pathogen susceptibility, viral infection

## Introduction

The recent Severe Acute Respiratory Syndrome Coronavirus 2 (SARS-CoV-2) pandemic is currently straining the global health system. Little is still known about this novel Coronavirus (CoV), despite the efforts of the scientific community worldwide. So far, analogies with the previous infamous outbreaks of Severe Acute Respiratory Syndrome (SARS) in 2003 and Middle East Respiratory Syndrome (MERS) in 2012, caused by other CoV strains, have offered some insight, but we are still sailing in uncharted waters.

**Figure 1:**
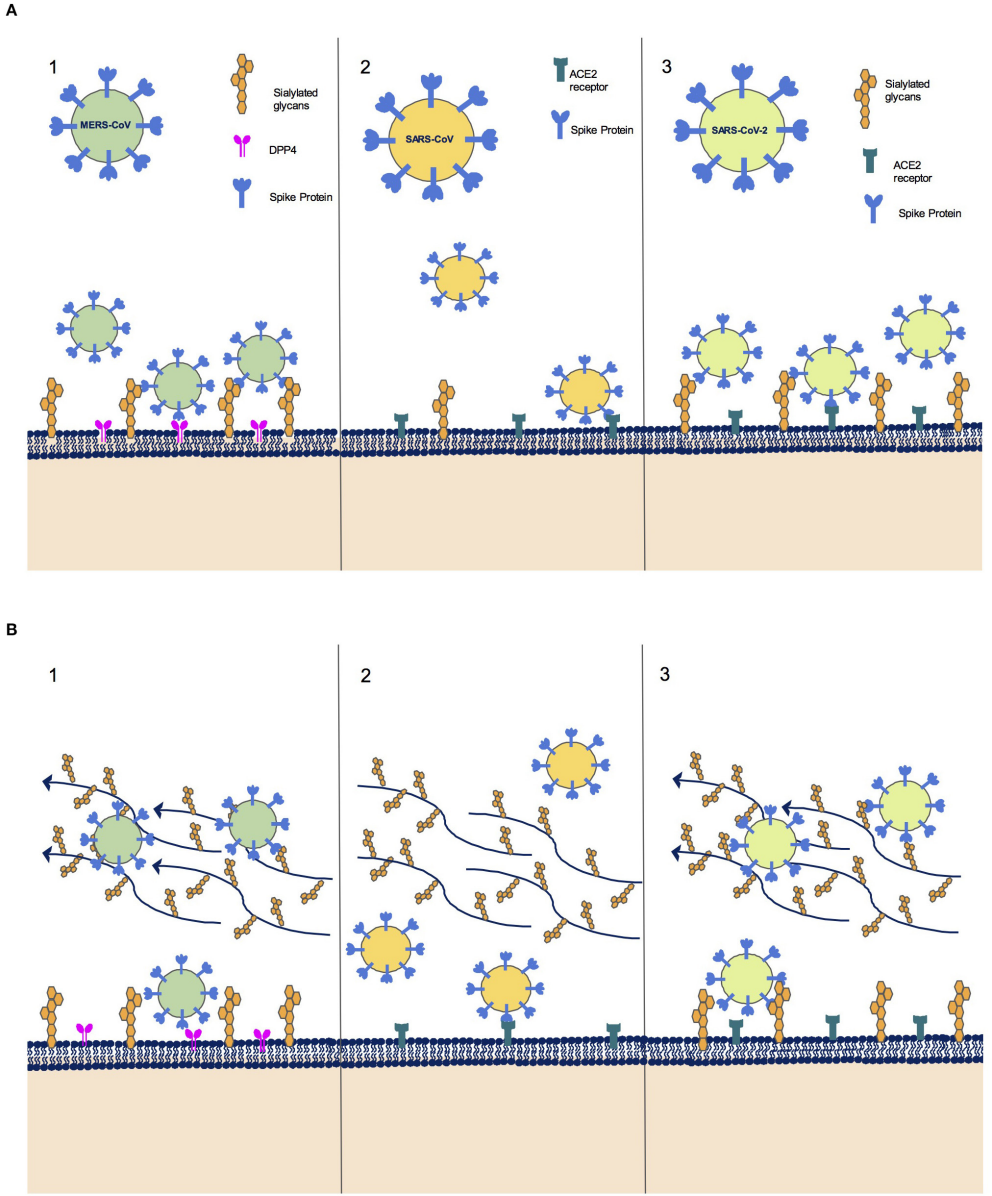
**(A)** Sialic acid recognition as an infection facilitator for Coronavirus strains. (1) MERS-CoV binds to non-acetylated sialoside receptors on the epithelial cells of the respiratory tract, promoting clustering and facilitating its binding to its receptor DPP4. (2) SARS-CoV binds to ACE2 receptor. (3) SARS-CoV-2 binds to ACE2 receptor, but a surface region in Spike protein is very similar to MERS-CoV spike sialic acid-binding region, suggesting a possible role of sialic acid recognition in infection initiation. **(B)** Sialic acid recognition as a host defense mechanism for Coronavirus strains. (1) MERS-CoV can bind to sialylated O-linked glycans covering mucins on mucosal cell surfaces, thus being trapped in the mucous layer and consequently eliminated through ciliary movement. (2) SARS-CoV passes through the mucous layer without being stopped by decoy alternative binding sites. (3) SARS-CoV-2 shares with MERS-CoV the sialic acid binding region of Spike protein, and could therefore bind to sialylated O-linked glycans similarly to MERS-CoV, thus possibly being eliminated through ciliary movement.

Although all CoV infections are initiated by the transmembrane spike (S) glycoprotein, a homotrimeric class I viral fusion protein, the binding site on the host cell surface differs among CoV strains (Figure 1A). MERS-CoV weakly binds to non-acetylated sialoside attachment receptors on epithelial cells of the respiratory tract, promoting clustering and facilitating its binding to its receptor dipeptidyl peptidase-4 (DPP4) (1). The novel SARS-CoV-2, despite having evolved independently, shares with the previous SARS-CoV the cell receptor for Angiotensin Converting Enzyme 2 (ACE2) (2). However, a novel study by the Italian Institute of Technology (3) suggests that there is an *in-silico* evidence that, in addition to ACE2, certain sialic acids on the cell surface may act as additional receptors for binding sites of the S protein of SARS-CoV-2, thus playing a role in the pathogenicity and epidemiology of the associated disease, as it has already been demonstrated for MERS-CoV. Sialic acids could therefore be used by SARS-CoV-2 as attachment receptors on the epithelium of the respiratory tract, promoting SARS-CoV-2 clustering, as already known for MERS-CoV (1). As a result, virus-ACE2 binding could be facilitated. Furthermore, a recent research by Vandelli and colleagues explored the structural properties of SARS-CoV-2 strains through computational approaches, and found that the ACE2 binding site is conserved among strains, whereas the potential SARS-CoV-2-sialic acid binding domain is highly variable, as reported in MERS-CoV. This variability could result in different binding affinities of SARS-CoV-2 strains for cellular sialic acids, possibly explaining the broad range of host-immune responses in the human population (4).

## The Human Sialome

### The Sialome Evolution

The human *sialome*, i.e., the broad variety of sialic acid compounds in the human body, has been hypothesized to be the result of genomic changes occurred under the selective impulse of an alleged pandemic event, roughly 3 million years ago, provoking the so-called *sialoquake*.

Varki has well-described the paramount role of sialic acids in pandemic events. Observing that humans only synthesize N-Acetyl-Neuraminic Acid (NeuAc), whilst in humans' closest evolutionary relatives both NeuAc and N-Glycolylneuraminic acid (NeuGc) can be found, Varki postulated that some unknown pathogen caused the evolutionary selection of a specific variant of the enzyme CMP-N-acetylneuraminic acid hydroxylase (CMAH), responsible for a key reaction in the synthesis of NeuGc. This unknown pathogen, having a high affinity for NeuGc binding, caused a pandemic event that exerted an evolutionary pressure, leading to the deletion of one exone in the CMAH gene, that made the enzyme incapable of synthesizing NeuGc, while the synthesis of NeuAc remained untouched. Although differing only by one oxygen atom, the altered proportions of NeuAc and NeuGc (loss of the latter and relative increase of the former), allegedly altered human susceptibility to pathogens, protecting them from NeuGc-binding pathogens, while exposing them to NeuAc-binding ones (5). This difference in neuraminic acid synthesis between humans and other mammals still has relevant consequences. Although MERS-CoV receptor, DDP4, does not differ between humans and dromedaries or horses, MERS-CoV's natural hosts, the latter appear to be resistant to experimental MERS infection, suggesting that other factors are involved in host susceptibility. Noting that MERS-CoV has a binding site of high selectivity for NeuAc that excludes NeuGc, and that NeuAc is less represented in horses lower airways, it could be speculated that the differences in sialoglycomes among species affect host susceptibility and tissue tropism (1).

### The Antiviral Protective Role of the Sialome

Sialic acid viral recognition has been long known to be a virulence factor for various pathogens (6). However, the sialome exerts also a protective effect against viral infections (Figure 1B). As a host defense mechanism, sialylated O-linked glycans covering mucins on mucosal cell surfaces provide a large layer of sialylated residues that acts as a barrier, preventing pathogens from entering the cell by offering a decoy alternative binding site.

*In vivo* studies have demonstrated that knockout Muc1^−/−^ mice (i.e., genetically modified to lack mucin 1) challenged with H1N1 Influenza A virus reach maximal viral titers earlier and with greater inflammatory response using equivalent viral challenge titers, compared to their wild-type counterparts (7). As further proof of the protective role of sialylated compounds, it is worth mentioning how concentration of oligosaccharides (HMOs), glycosylated components of human breast milk, in HIV-positive women correlates with reduced HIV transmission to the nursling through breastfeeding. Moreover, it is well-known how HMOs interfere with viral glycoprotein recognition of Norovirus and Rotavirus, playing a pivotal anti-viral role, which, in addition to their positive effect on neurodevelopmental outcomes, has justified their supplementation in infant formulas (8). However, HMOs' potential role in preventing, limiting or modulating SARS-CoV-2 infection has not been explored, yet.

### Sialome Age and Sex-Related Modifications

Like most of the human body components, the sialome undergoes aging-dependent deleterious processes as well. Sialylation is a modification through which a sialic acid unit is added at the end of an oligosaccharide chain in a glycoprotein. Among sialylated serum proteins, IgG-Fc terminal glycan sialylation has been extensively studied for its importance in inflammatory diseases, either autoimmune or infectious, due to the modulation of pro- and anti-inflammatory cascades by aglycosylated and glycosylated IgGs, respectively. Recent studies have identified an age-related accumulation of aglycosylated IgGs, which is linked to a pro-inflammatory status, typical of the elderly. Moreover, elderly patients exhibit a lower sialic acid content in saliva compared to children, confirming that sialylation processes decrease all over the body with aging. Similarly, sialome seems to be affected by the body's hormonal asset, in that estrogens upregulate antibody sialylation, determing an anti-inflammatory effect, whilst a decrease in estrogen levels, as seen in menopause, leads to lower sialylation activity. In line with these findings, pregnancy seems to be a “highly sialylated status,” which may reflect the well-known reduced incidence of inflammatory or autoimmune disease flares during this period of time. Interestingly, trans-placental passage of maternal glycosylated IgGs results in the anti-inflammatory IgG profile of new-borns, with glycosylated IgG levels that decrease over the years, until they reach adult levels (9). Applying these findings to the current pandemic situation, it could be interesting to assess whether a low-sialylated environment in men and elderly could play a role in SARS-CoV-2 infection both by favoring infection initiation, due to the low-grade sialylation of the defensive respiratory mucus, and by enhancing the inflammatory state caused by the subsequent cytokine storm, partly explaining the higher prevalence and severity of COVID-19 in male and older patients and the diminished aggressiveness in pregnant women and new-borns (10).

## Discussion

At the present level of knowledge, it cannot be confirmed nor excluded that COVID-19 clinical manifestations differ according to individual differences in sialic acid expression on cell surfaces. However, what is already known about the human sialome and CoV strains allows us to postulate that the epidemiologic characteristics of COVID-19 (greater severity in male and older individuals) may be partially explained by the sex and age-related differences of sialome among humans.

Despite multiple data generated using anti-viral repurposed drugs, to date neither a vaccine nor any effective specific treatment are available. Even anti-inflammatory drugs have not obtained regulatory approvals to be used to fight the cytokine storm causing the Acute Respiratory Distress Syndrome (ARDS), the most severe expression of Acute Lung Injury (ALI). Prevention as well has been limited by the extreme contagiousness of SARS-CoV-2 and, to this day, the most effective measure has been general lockdown. A deeper comprehension of the role of human sialome in this pandemic could contribute to the development of preventive strategies targeted at the most vulnerable subjects, maybe even considering upregulating sialylation through the supplementation of exogenous synthetic sialylated compounds, as it has already been done in other contexts and for other purposes in infant formulas. Indeed, sialic acids could be provided to patients within a combined therapy to reduce inflammation and viral load, that ultimately result in the COVID-19 associated respiratory distress syndrome, the most severe COVID-19 expression, able to determine more than 50% of COVID-19 associated deaths.

In conclusion, we think that, altogether, data provided here should help to consider sialic acids as an important game-changer in the SARS-CoV-2 infection, since there are still several virus-cell interaction aspects that need to be discovered. Due to SARS-CoV-2′s low selective-pressure, we aren't currently facing a *quake* like that of 3 million years ago; however, every step made now toward a better comprehension of human susceptibility to pathogens would nonetheless have a paramount role in facing emerging global health threats.

## Author Contributions

DM conceived the presented idea and wrote the manuscript with support from AC. MG, CP, and FM critically reviewed and revised the manuscript. All authors read and approved the final manuscript as submitted.

## Conflict of Interest

The authors declare that the research was conducted in the absence of any commercial or financial relationships that could be construed as a potential conflict of interest.
